# Coordination polymer-forming liquid Cu(2-isopropylimidazolate)†

**DOI:** 10.1039/d2sc03223f

**Published:** 2022-09-12

**Authors:** Teerat Watcharatpong, Taweesak Pila, Thana Maihom, Tomohiro Ogawa, Takuya Kurihara, Koji Ohara, Tadashi Inoue, Hiroyasu Tabe, Yong-Sheng Wei, Kanokwan Kongpatpanich, Satoshi Horike

**Affiliations:** Department of Materials Science and Engineering, School of Molecular Science and Engineering, Vidyasirimedhi Institute of Science and Technology Rayong 21210 Thailand horike@icems.kyoto-u.ac.jp; Department of Chemistry, Faculty of Liberal Arts and Science, Kasetsart University Kamphaengsaen Campus Nakhon Pathom 73410 Thailand; Institute for Integrated Cell-Material Sciences-VISTEC Research Center, Institute for Advanced Study, Kyoto University Yoshida-Honmachi, Sakyo-ku Kyoto 606-8501 Japan; Diffraction and Scattering Division, Japan Synchrotron Radiation Research Institute (JASRI) Sayo 679-5198 Hyogo Japan; Department of Macromolecular Science, Graduate School of Science, Osaka University Toyonaka Osaka 657-0043 Japan; Department of Synthetic Chemistry and Biological Chemistry, Graduate School of Engineering, Kyoto University Katsura, Nishikyo-ku Kyoto 615-8510 Japan

## Abstract

The structure of the melt state of one-dimensional (1D) coordination polymer crystal Cu(isopropylimidazolate) (melting temperature *T*_m_ = 143 °C) was characterized by DSC, variable temperature PXRD, solid-state NMR (SSNMR), viscoelastic measurements, XAS, and DFT-AIMD calculations. These analyses suggested “coordination polymer-forming liquid” formation with preserved coordination bonds above *T*_m_. Variable chain configurations and moderate cohesive interaction in adjacent chains are the keys to the rarely observed polymer-forming liquid. The melt structure is reminiscent of the common 1D organic polymer melts such as entanglement or random coil structures.

## Abstract 2

The melt state of coordination polymer crystals is composed of macromolecular-chain assemblies without coordination bond breaking. The coordination-polymer-forming liquid provides various morphologies, including spun fibers.

## Graphical abstract

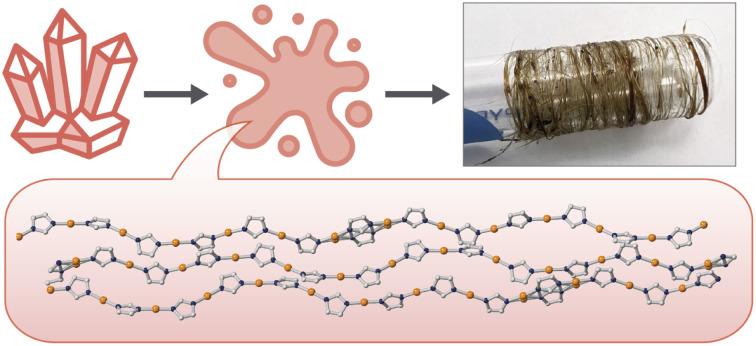

## Introduction

In the molten state of representative one-dimensional (1D) organic polymers such as polyethylene, polypropylene, and so on, they take random coil structures and form entanglement networks if their molar mass is high enough.^1^ Isotropic and flexible structures are possible due to the strong covalent bonds with sp^3^ carbons and resulting high internal freedom of the polymer chains. Understanding the structure and dynamics of organic polymer melts has been essential for materials engineering such as fibers by melt spinning, and mechanically tough films by oriented crystallization of melts.^2^

Recently, crystal melting and glass formation of coordination polymers (CPs) or metal–organic frameworks (MOFs) have gained much attention.^3–12^ The CP/MOF glasses are regarded as a new family of glass types,^13^ with unique non-crystalline properties including gas permeability, conductivity, phase switching, emission, and encapsulation.^11,14–19^ Although there are many studies about the structure of these glasses, the structural investigation of the melt state is yet limited. In the case of three-dimensional (3D) frameworks, including [Zn(imidazolate)_1.75_(benzimidazolate)_0.25_] (ZIF-62),^20^ the bridging ligands facilitate the partial breaking of coordination bonds upon melting, and thus promote a lower fusion of enthalpy Δ*H*_fus_.^9,21,22^ For example, the reduction in the average coordination number of 3D MOF (ZIF-4, [Zn(imidazolate)_2_]) upon melting is 10–18% by MD simulations.^21,23^ The strength of coordination bonds is smaller than that of covalent bonds, and the network flexibility of 3D CP/MOFs is smaller than that of sp^3^ carbon-based organic polymers in general.

1D CPs are structurally more related to the common 1D organic polymers, but there is no report that the melt state is composed of polymeric structures, because of the same reasons as for 3D frameworks. It is a challenge to find CPs whose melt state is composed of polymeric structures without bond breaking. The polymer-forming melt behavior is relevant to organic polymers, and it would open various ways of materials fabrication of CP/MOF fibers and films, and engineering such as injection molding.

## Results and discussion

**Fig. 1:**
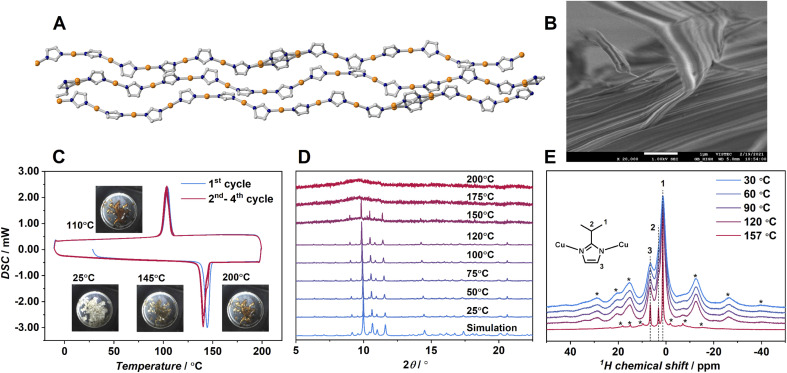
(A) Crystal structure. Cu: orange; C: gray; N: dark blue and (B) SEM image of 1. Isopropyl groups are omitted. (C) DSC profiles on the first cycle (sky blue) and the second to fourth cycles (red) of 1. 10 K min^−1^ under N_2_ flow. (D) VT-PXRD of 1. Wavenumber was *λ* = 0.99998 Å. (E) ^1^H magic-angle spinning solid-state NMR of 1 at temperatures of 30, 60, 90, 120, and 157 °C. Asterisk denotes spinning sidebands.

To find the coordination polymer-forming melt, we employed 1D CPs having a variety of polymer configurations with strong coordination bonds. We selected a 1D CP Cu(ipim) (1, Hipim = 2-isopropylimidazole).^24^ The previous paper reported the crystal structure and differential scanning calorimetry (DSC) of 1. The 1 shows an endothermic peak at 143 °C in the DSC upscan suggesting the crystal melting. The unique feature of the crystal structure of 1 is that the unit cell contains 6.5 crystallographically independent Cu(ipim) units to form wavy 1D chains (Fig. 1A).

Each chain is packed in parallel to one another through van der Waals interactions. One of the imidazolate rings shows a two-site disorder. The crystal structure has eight different angles of N–Cu–N, ranging from 172.2 to 180.0° (Fig. S10A).† The dihedral angle between adjacent imidazolato rings also varies significantly. These structural features suggest that the 1D chains in 1 form a variety of conformations to enlarge the fusion of entropy Δ*S*_fus_, although strong metal–azolate bonds would preserve the polymeric structure. According to the literature, the crystalline powder of 1 was synthesized and confirmed by powder X-ray diffraction (PXRD).^24^ The SEM image of 1 (Fig. 1B) showed a fibrous morphology reflected by the anisotropic 1D chain assembly. Thermogravimetric analysis (TGA) of 1 (Fig. S1†) showed no weight loss up to 340 °C indicating that the composition of 1 does not change up to this temperature. DSC of 1 (Fig. 1C) with *in situ* sample imaging showed a sharp endothermic peak onset at 143 °C, indicating the crystal melting of 1. Hereafter, we call the melt state of 1 as 1-melt. Δ*H*_fus_ calculated by DSC is 6.2 kJ mol^−1^ and the entropy of fusion Δ*S*_fus_ is 14.9 J mol^−1^ K^−1^. They are comparable to those of ZIF-62 (Δ*H*_fus_ = 3.5 kJ mol^−1^, Δ*S*_fus_ = 5.0 J mol^−1^ K^−1^) which is composed of strong Zn^2+^–N bonds.^12,25^ In the cooling process of 1-melt in DSC, a sharp exothermic peak was observed at 112 °C (Δ*H*_crys_ = −5.0 kJ mol^−1^, Δ*S*_crys_ = −13.8 J mol^−1^ K^−1^). The peak corresponds to the crystallization of 1-melt to 1, as we observed an identical PXRD pattern for the cooled sample at 25 °C.

The facile crystallization of 1-melt to 1 by gentle cooling is unusual among the other reported CP/MOFs.^12^ Most of the melt states of the reported CP/MOFs transform to the glassy state by cooling because of high viscosity, and one example has clearly shown the crystallization from the melt so far.^9^ We conducted a rapid quenching of the 1-melt by soaking in liquid N_2_, but it showed crystallization as confirmed by PXRD. Four heating/cooling cycles of DSC are shown in Fig. 1C, which show the same traces for all the cycles. PXRD of 1 at 25 °C after four cycles of DSC measurements was identical to that of 1 (Fig. S2).† Thus, the crystal melting and recrystallization behavior are reversible.

Variable temperature (VT)-PXRD was performed to investigate the structural transformation from 1 to 1-melt (*λ* = 0.99998 Å, Fig. 1D). PXRD patterns of 1 in the range of 25 to 120 °C were unchanged. In contrast, we observed a mixed pattern of crystalline and amorphous states at 150 °C which is just above the melting temperature *T*_m_ (143 °C) by DSC. This indicates the coexistence of amorphous (melt) and crystalline states. Although we should take different temperature ramping rates of DSC and PXRD into account, the mixed phase at near *T*_m_ would be intrinsic for 1 and it reminds us of the imperfect crystallinity of common organic polymers. PXRD patterns above 175 °C suggest that the sample totally transformed to 1-melt. VT ^1^H magic-angle spinning solid-state NMR was performed to study the structure and dynamics of 1 and 1-melt (Fig. 1E). Proton peaks at 1.4, 3.2, and 6.6 ppm correspond to the –CH_3_, alkyl CH and N–CH, and NH groups of the ipim ligand, respectively. Each peak is broadened at 30 °C, and spinning side-bands are observed in the broad frequency region, suggesting the low mobility of the structure. The spectra were unchanged at 120 °C indicating that the structure and dynamics of 1 are intact as supported by VT-PXRD. The spectrum of 1-melt at 157 °C showed sharp peaks in addition to the broad peaks. The sharp peaks indicate the enhanced mobility of the ligands due to the crystal melting. The broad residual peaks are ascribed to micro-crystals remaining above *T*_m_, which is also consistent with VT-PXRD. The sharp peaks have spinning side-bands, suggesting the presence of weak anisotropic nuclear spin interactions (*i.e.*^1^H dipolar interaction and/or chemical shift anisotropy). This means that the dynamics of the ligands in 1-melt are anisotropic even under the melt state.

**Fig. 2:**
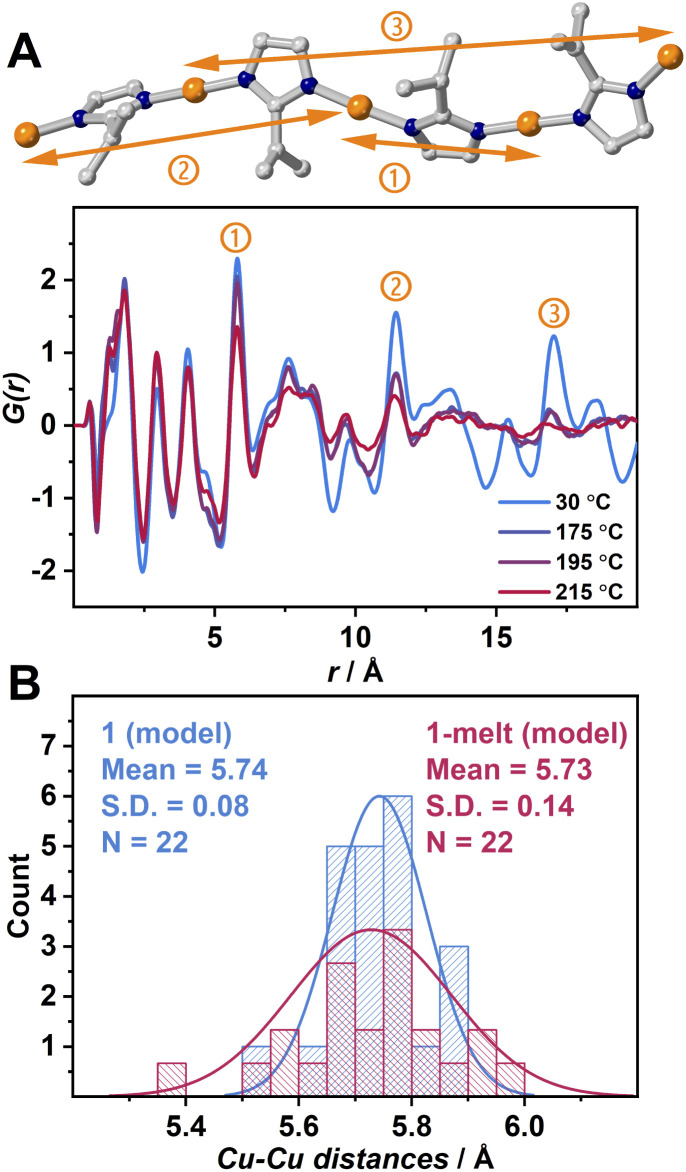
(A) Partial crystal structure of 1 showing the intrachain Cu–Cu correlations at approximately ① 6.10, ② 11.6, ③ 17.1 Å and radial distribution function of 1 at 30 °C and 1-melt at 175, 195 and 215 °C. (B) Distribution plots of Cu–Cu distances of 1(model) (blue) and 1-melt(model) (red) generated from MD simulation. S.D. is the standard deviation, and N is the number of the population.

X-ray scattering measurements were performed to obtain pair distribution function (PDF) data of 1 and 1-melt at various temperatures (Fig. 2A). A simulated PDF profile from a single crystal X-ray structure of 1 (Fig. S4A and S5†) was used to assign the peaks. The dominant components of three prominent peaks at 6.10, 11.6, and 17.1 Å observed at 30 °C are the nearest neighbor, second-neighbor, and third-neighbor correlations of two intrachain Cu^+^ ions respectively (① ∼ ③). We also found the correlations of N–N overlap in the peaks ① and ② though the intensities are lower than those of Cu–Cu. This is because N atoms are the first neighboring atoms and coordinate with Cu^+^ ions in a linear fashion. These three peaks ① ∼ ③ in *G*(*r*) are measured at 175 and 195 °C which are above *T*_m_ are shown. The peak intensities and position of the nearest intrachain Cu–Cu neighbor (①) at 175 and 195 °C are unchanged as those at 30 °C. This suggests that the Cu-ipim-Cu bridges remain above *T*_m_. The intensities of the two peaks of second/third neighbors ② and ③ at 175 and 195 °C are reduced compared with the case at 30 °C, suggesting lower structural periodicity due to thermal activation.

To distinguish the contributions of both intrachain and interchain Cu–Cu to the PDF results, we generated a PDF profile by creating a modified crystal structure of 1 leaving only the atoms forming a single chain to exclude the interchain correlations (Fig. S4B).† The 7.45 and 13.5 Å peaks corresponding to Cu–Cu are not observed. This suggests that these peaks are attributed to interchain Cu–Cu. In Fig. 2A, the peak at 7.45 Å is sufficiently intense at 175 °C, while the 13.5 Å peak is significantly attenuated. At 215 °C, both peaks almost disappear. Considering that peaks ① and ② retain some intensity at 215 °C, it is suggested that the correlations in interchain Cu–Cu tend to be smaller than those in intrachain Cu–Cu upon heating. We also observed the shift of these two peaks ② and ③ to be shorter upon heating, and this is because of the higher degree of conformation. The presence of these correlation peaks above *T*_m_ indicates that the 1-melt is composed of coordination polymers having sufficient molecular weight.

Theoretical calculations were conducted to understand the structural transformation upon melting. DFT was applied to structural optimization of the crystal structure of 1, and then *ab initio* molecular dynamics (AIMD) simulations were executed at 25 and 175 °C named as 1(model) and 1-melt(model), respectively (Fig. S6–S8).† We found that the intramolecular reduced pair distribution function *G*(*r*) produced from 1-melt(model) is similar to that of 1 (Fig. S9†) by observing peaks at 6.1, 11.6, and 17.1 Å in Fig. 2A. This indicates that the 1-melt(model) successfully describes the structure in 1-melt. A distribution of N–Cu–N bond angles of 1 (model) and 1-melt(model) (Fig. S13†) shows that the larger standard deviation in 1-melt(model) indicates the greater structural conformation of the 1-D chain upon melting. Fig. 2B shows the distribution of Cu–Cu distances in 1(model) and 1-melt(model). 1-melt(model) has a broader distribution and it also supports the higher disorder of the melt state with a 1D chain configuration.

We measured variable temperature X-ray absorption spectroscopy (XAS) at 25, 175, 300, 90, and 25 °C to quantify the coordination numbers (CNs) of Cu^+^ using 1(model) and 1-melt(model). The results are summarized in Table S1.† The CNs are estimated to be 1.97 at 175 °C and 1.79 at 300 °C. This suggests that the CN is almost unchanged above *T*_m_ and this supports the discussion about the retention of the 1D chain structures above *T*_m_ without bond breaking. The lower CN at 300 °C increases upon cooling and it is 1.98 at 25 °C. This indicates that the coordination bonds of N–Cu reform below *T*_m_ because of less thermal activation.

**Fig. 3:**
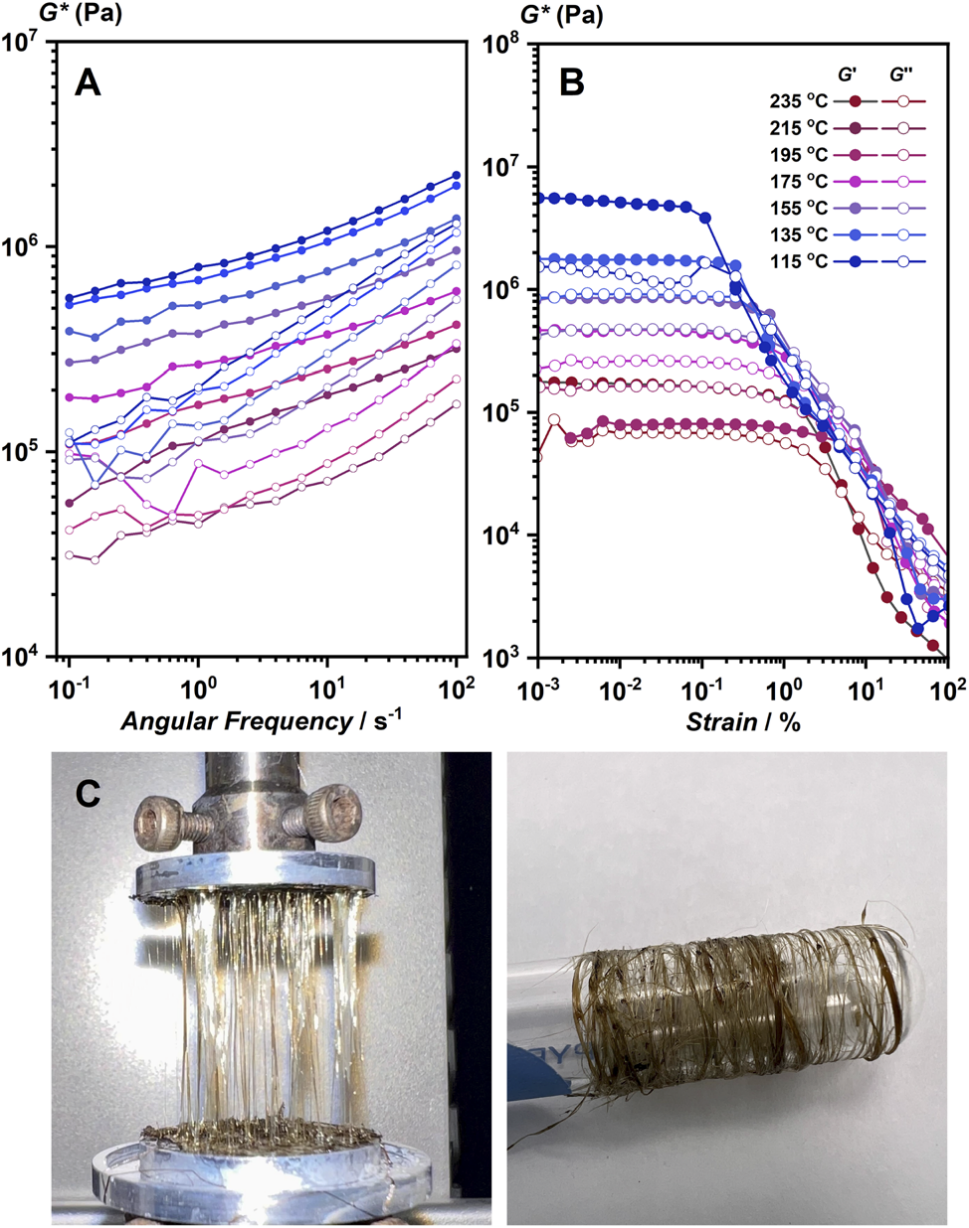
(A) Frequency sweep and (B) strain sweep viscoelastic measurements of 1 from 235 to 115 °C under N_2_ flow. (C) Fibers of 1 prepared by a melt-quench process by use of hot press and collected by spinning around the glass tube.

Viscoelastic properties of 1 and 1-melt were studied further to support the discussion of the structure of 1-melt. The samples were loaded into a rheometer at 250 °C and cooled stepwise. The strain sweep and frequency sweep test results under the isothermal conditions are shown in Fig. 3A and B, respectively. In the melting state, the real part of the complex modulus, *G*′, is approximately 10^6^ Pa, and did not depend on the frequency (Fig. 3A). This indicates that the molten state has a high viscosity and requires a certain external force to deform. This property suggests the elasticity and the existence of the network structure. The storage modulus of the compound should be much lower in case the structure does not remain as a 1D network. In addition, careful observation revealed that the moduli at different temperatures could superpose with each other with a horizontal shift and make a composite curve (master curve, Fig. S14),† which is called the time–temperature superposition principle (method of reduced variables). These features are characteristic of ordinary flexible organic polymeric systems such as polystyrene. On the other hand, the linear viscoelastic regime was less than 1% and decreased slightly with decreasing temperature (Fig. 3B). The stronger strain dependence of modulus than that in flexible polymers suggests that the network in the molten state is an entangled network of stiff chains. Another possibility is that the system contains an aggregate of solid particles.^26^ These observations lead to the proposed model that there are both microcrystalline and melt phases in this temperature region, which coincides with PXRD and SSNMR results. The rheological characterization also suggested that the structure of 1-melt is composed of long linear molecules. We prepared fiber-shaped samples from hot-pressed 1-melt by use of a rheometer machine. The fibrous sample was collected by pulling up 1-melt (Fig. 3C), and we can collect these fibers by use of a glass tube. The fiber sample showed crystalline PXRD the same as the simulated pattern of the crystal structure of 1 (Fig. S15†).

## Conclusions

The work elucidated that the melt state of 1D CP Cu(ipim) (Hipim = 2-isopropylimidazole) is composed of polymeric networks without significant coordination bond breaking. DSC, variable temperature PXRD, SSNMR, XAS, viscoelastic measurements, and DFT-AIMD calculations suggested that the “polymer-forming liquid” is the most probable structure above *T*_m_. It is essentially different from 3D MOF cases which require bond-breaking upon melting. Variable chain configurations attributed to the linear coordination geometry and moderate cohesive interaction in adjacent chains are the keys to the polymer-forming liquid. The melt structure is reminiscent of the common 1D organic polymer melts, and the utilization of the polymer melt would expand the materials engineering including oriented, mechanically tough films or fibers.

## Author contributions

T. W. and T. P.: data curation, investigation, validation, writing – original draft. T. M. and T. O. and T. K. and K. O. and T. I. and H. T. and Y. -S. W.: formal analysis, investigation, resources, validation, writing – review & editing. K. K. and S. H.: conceptualization, funding acquisition, project administration, supervision, writing – review & editing. All authors critically reviewed and revised the manuscript draft and approved the final version for submission.

## Conflicts of interest

There are no conflicts to declare.

## Supplementary Material

SC-013-D2SC03223F-s001
