# Supplementary material for: Coordination polymer-forming liquid Cu(2-isopropylimidazolate)
Source: Chem Sci. 2022 Sep 12;13(38):11422–6. doi: 10.1039/d2sc03223f (PMC9533396; doi:10.1039/d2sc03223f)
Supplement: SC-013-D2SC03223F-s001 [file SC-013-D2SC03223F-s001.pdf]

Electronic Supplementary Material (ESI) for Chemical Science.
This journal is © The Royal Society of Chemistry 2022

Supporting Information for

Coordination polymer-forming liquid Cu(2-isopropylimidazolate)

Teerat Watcharatpong,[a] Taweesak Pila,[a] Thana Maihom,[b] Tomohiro Ogawa,[c] Takuya Kurihara,[c] Koji Ohara,[d] Tadashi Inoue,[e] Hiroyasu Tabe,[c] Yong-Sheng Wei,[c] Kanokwan Kongpatpanich,[a] Satoshi Horike[a,c,f]

[a] Department of Materials Science and Engineering, School of Molecular Science and Engineering, Vidyasirimedhi Institute of Science and Technology, Rayong 21210, Thailand

[b] Department of Chemistry, Faculty of Liberal Arts and Science, Kasetsart University, Kamphaengsaen Campus, Na-khonpathom 73410, Thailand

[c] Institute for Integrated Cell-Material Sciences-VISTEC Research Center, Institute for Advanced Study, Kyoto University, Yoshida-Honmachi, Sakyo-ku, Kyoto 606-8501, Japan

[d] Diffraction and Scattering Division, Japan Synchrotron Radiation Research Institute (JASRI), Sayo, Hyogo 679-5198, Japan

[e] Department of Macromolecular Science, Graduate School of Science, Osaka University Toyonaka, Osaka 657-0043, Japan

[f] Department of Synthetic Chemistry and Biological Chemistry, Graduate School of Engineering, Kyoto University, Katsura, Nishikyo-ku, Kyoto 615-8510, Japan

General information

All materials and solvents for syntheses were available commercially and used without any purification.

Synthesis: 1 was synthesized by using a solvothermal reaction reported in the literature.[1] 1.21 g of $Cu(NO_3)_2 \cdot 3H_2O$ and 1.10 g of 2-isopropylimidazole were dissolved in 30 mL of 25% aqueous ammonia by stirring for 15 min under air in a vial. The obtained solution was then transferred and sealed in 100 mL Teflon-lined vessel. Teflon-lined vessel was heated in an oven at 160 °C for 80 h. After cooling down to room temperature, pale yellow crystals were collected by filtration and washed with ethanol for 3 times. The received product was dried under N_2 for overnight and kept inside an Ar filled glove box.

Thermal analyses: Thermogravimetric analysis (TGA) was performed using a Rigaku TG8120 under flowing N_2 (50 mL min^{-1}) with heating rate of 10 K min^{-1}. Differential scanning calorimetry (DSC) were carried out using a Hitachi DSC7020 under flowing N_2 (50 mL min^{-1}) with heating rate of 10 K min^{-1}.

Dynamic viscoelastic measurements: These were conducted by two rheometers with parallel plate (ARES G2 and Discovery Hybrid Rheometer HR20, TA instruments) to determine the storage and loss moduli $G'(\omega)$ and $G''(\omega)$ at various angular frequencies ω. The instrument compliance was corrected before the measurements.[2-3] The dynamic strain amplitude sweep experiments were also conducted to ensure the linearity of the viscoelastic response. All the measurements were performed N_2 atmosphere.

X-ray measurements: Powder X-ray powder diffraction (PXRD) data were collected on a RIGAKU MiniFlex600 and SmartLab SE diffractometers both with CuKα anode (λ = 1.540598 Å). Variable temperature X-ray powder diffraction data were recorded using BL02B2 beamline at Super Photon ring-8 GeV (SPring-8, Hyogo, Japan, λ = 0.99998 Å) in temperature range RT to 200 °C. X-ray total scattering measurements were performed at SPring-8 on BL04B2 beamline. The samples were sealed in a glass capillary under Ar and the data was collected at 30, 175, 195, and 215 °C. A large Debye−Scherrer camera with an imaging-plate-type detector and imaging plate converting the Q range from 0.5 to 20 $Å^{-1}$ was used with an incident beam (37.668 keV; λ = 0.32915 Å). PDFgetX3 program was employed for the correction of the data for Compton scattering, multiplicative contributions, and Fourier transformation. The simulated PDF pattern was generated by the use of the single crystal structure of **1** (CCDC No. 1061186) with the software CrystalMaker® X version 10.7.0. To generate the model crystal structure of **1** having no interchain interaction is described as follows. The structure in the super-cell was obtained by Materials Studio 2020 software package (Accelrys Software Inc.). Based on the single crystal structure of **1** (CCDC No. 1061186), the distance of the simplest repeating unit of the single chain was confirmed to be 73.028 Å. In order to keep the distance between all adjacent chains are larger than 20 Å, a unit cell by 20.000 × 20.000 × 73.028 $Å^3$ with $P1$ space group was created by using the "3D Atomistic Document" mode in the Materials Studio. The single chain comes from the crystal structure of **1** without any further structural optimization. Specifically, the simplest repeating structural fragment was extracted (copied) from a 4 × 4 × 5 packing unit cell of **1** and pasted into the above-created unit cell. The chain was adjusted to the center of the unit cell along the c-axis, finally giving the periodically extensible single coordination bond chain structure.

Cu K-edge X-ray absorption spectroscopy (XAS) including X-ray absorption near edge structure (XANES) and extended X-ray absorption fine structure (EXAFS) regions were performed in the transmission mode at BL14B2 beamline at SPring-8. Cu foil internal energy calibration was carried out before sample measurements. The sample was pelletized prior to the measurement. The temperature-dependent XAS study was performed by heating and cooling the sample under N_2 with a ramp rate of 2 °C min^{-1}. The XAS results were processed by

the Athena software, and the EXAFS fitting was done by using Arthemis software.[4] The coordination number (CN) of **1** at each temperature (25, 175, 300, 90 and 25 °C) was obtained from the EXAFS fitting employing the structural models of **1(model)** and **1-melt(model)** from Ab initio MD simulations. The S_0^2 obtained from the fitting of EXAFS data at 25 °C was employed for the fitting of CN of **1** at other temperature points.

Solid-state NMR: ^{1}H solid-state magic angle spinning nuclear magnetic resonance spectra) were recorded on a JEOL RESONANCE JNM-ECZ600R (JEOL RESONANCE Inc.) solid-state NMR spectrometer (600 MHz) at 14.1 T. Spinning rates were 8 kHz for measurements at 30-120 °C and 5 kHz for measurements at 157 °C.

Theoretical calculation: *Ab initio* MD simulations (AIMD) within the generalized gradient approximation (GGA) were performed with the Vienna Ab-initio Simulation Package (VASP).[5-6] The Perdew–Burke–Ernzerfhof (PBE)[7] with the empirical correction in Grimme's scheme (DFT-D3)[8] was employed to evaluate the self-consistent field (SCF) energy. To treat the localization of 3d-electrons more accurately, an on-site Hubbard term U−J[9] of 4.0 eV for Cu was applied.[10] The energy cut-off was set to 450 eV and the Brillouin zone was sampled by the single Γ-center k-point grid. The convergence thresholds for SCF convergence criterion were set 10^{-5} eV between SCF iterations. In our simulation, the NPT ensemble was performed with a timestep of 1 fs. The temperature was controlled by Nosé–Hoover thermostats. The equilibration run was followed by a production run of 5 ps, depending on the temperature. **1(model)** was constructed by using 2 × 1 × 1 supercell layer to model its packing in the crystalline phase, while **1-melt(model)** was constructed by using the sample supercell performing at 175 °C to represent its packing in the melting state. MD simulation results were visualized and analyzed using VMD software.[11]

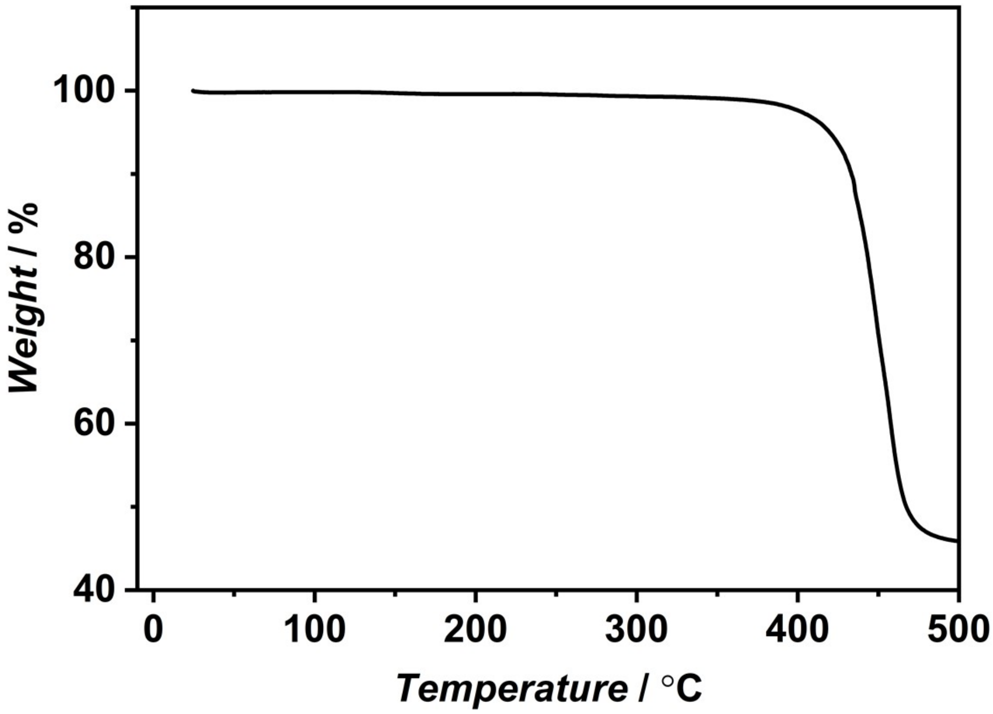

Figure S1. TGA profile of **1**. The scan rate was 10 °C min^{-1}.

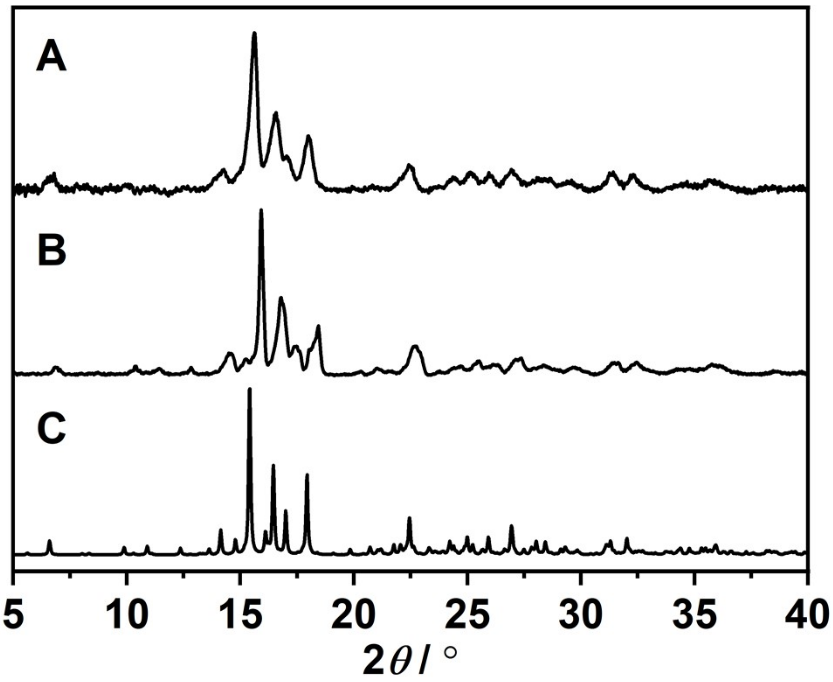

Figure S2. (A) PXRD patterns of **1** after four cycles of DSC measurement compared to (B) the as-synthesized sample of **1** and (C) the simulation from the crystal structure of **1** (λ = 1.540598 Å).

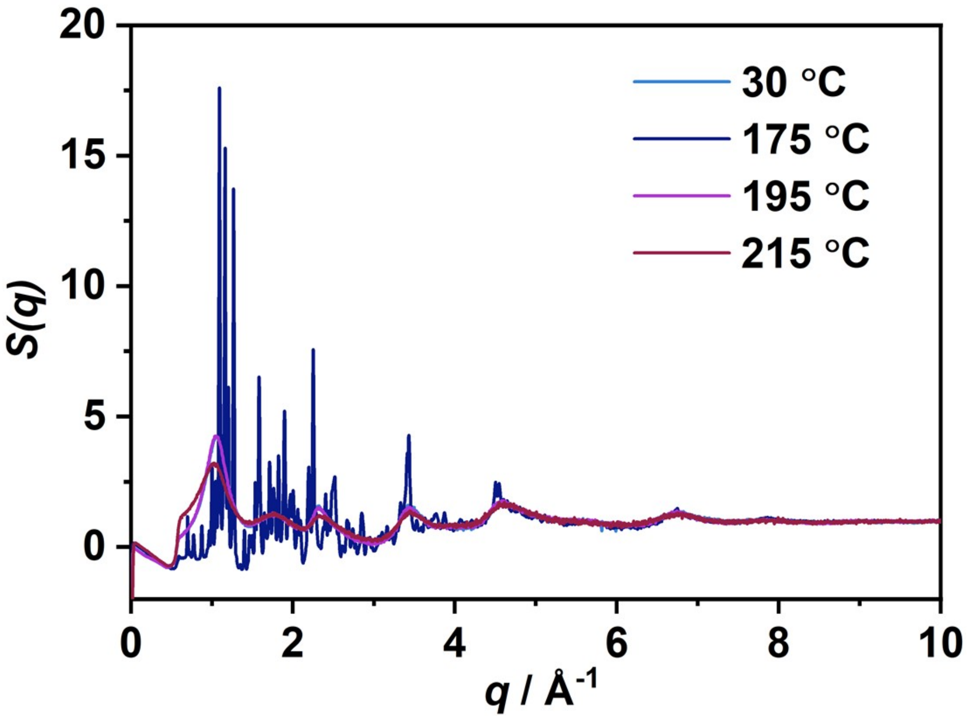

Figure S3. Structure function $S(q)$ of **1** in the temperature range of 30 to 215 °C.

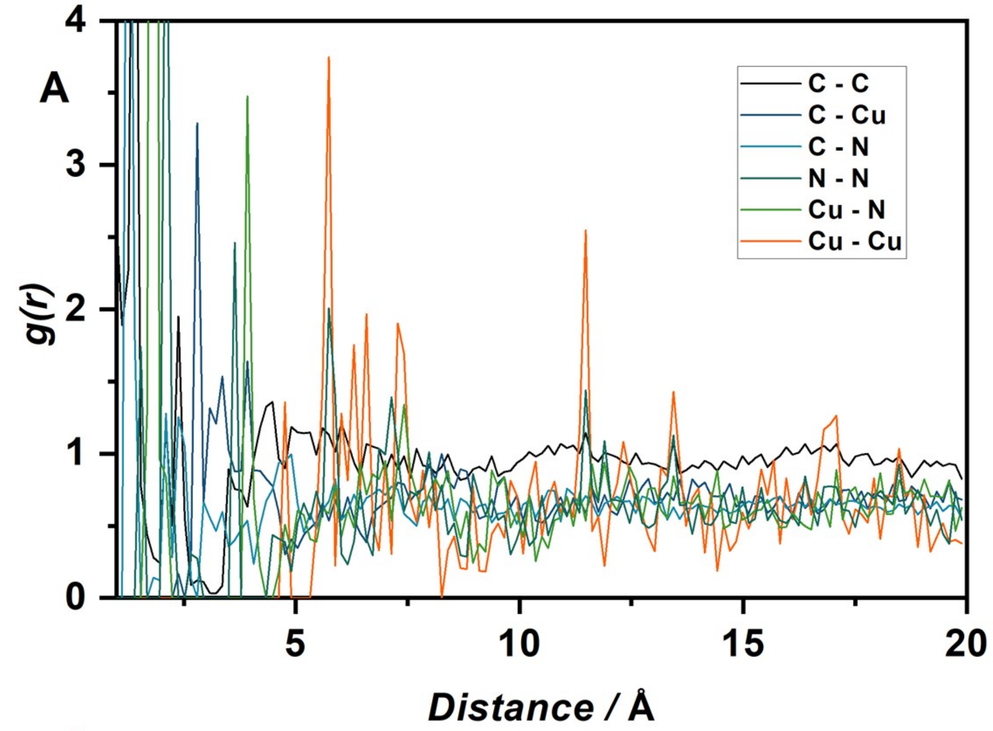

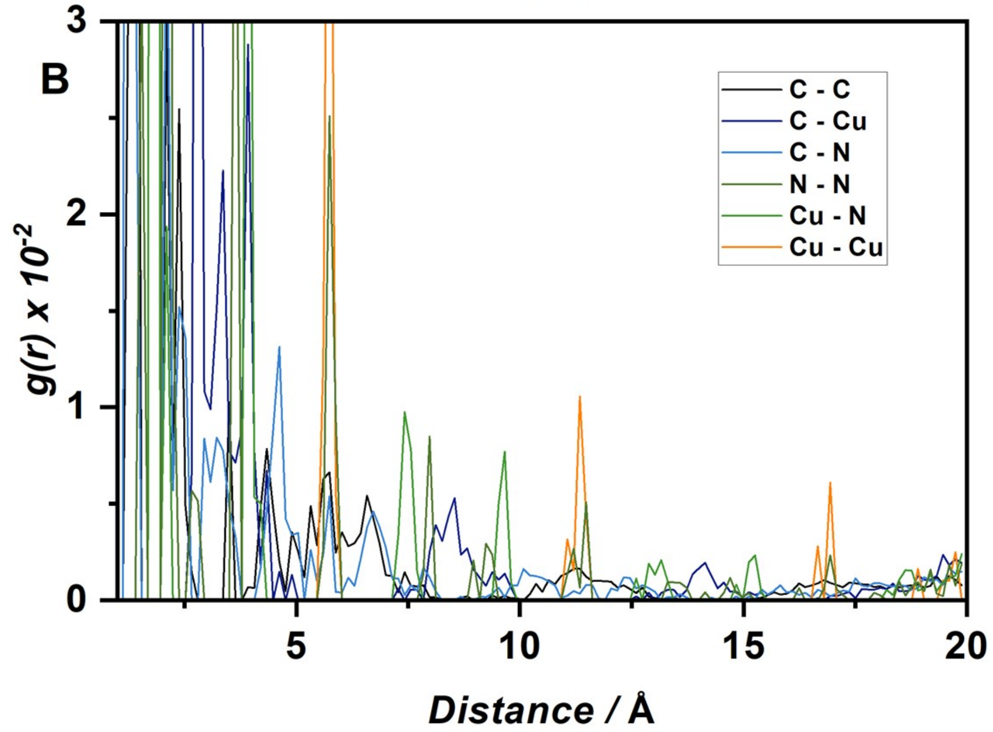

Figure S4. (A) Simulated partial distribution function (*g(r)*) of **1** generated from the single crystal X-ray structure. (B) Simulated partial distribution function (*g(r)*) of **1** generated from the modified single crystal X-ray structure which eliminates the interchain interaction. Details of preparation of this structural model was described at the **General information** part in above. Both PDF simulations are generated by the CrystalMaker software.

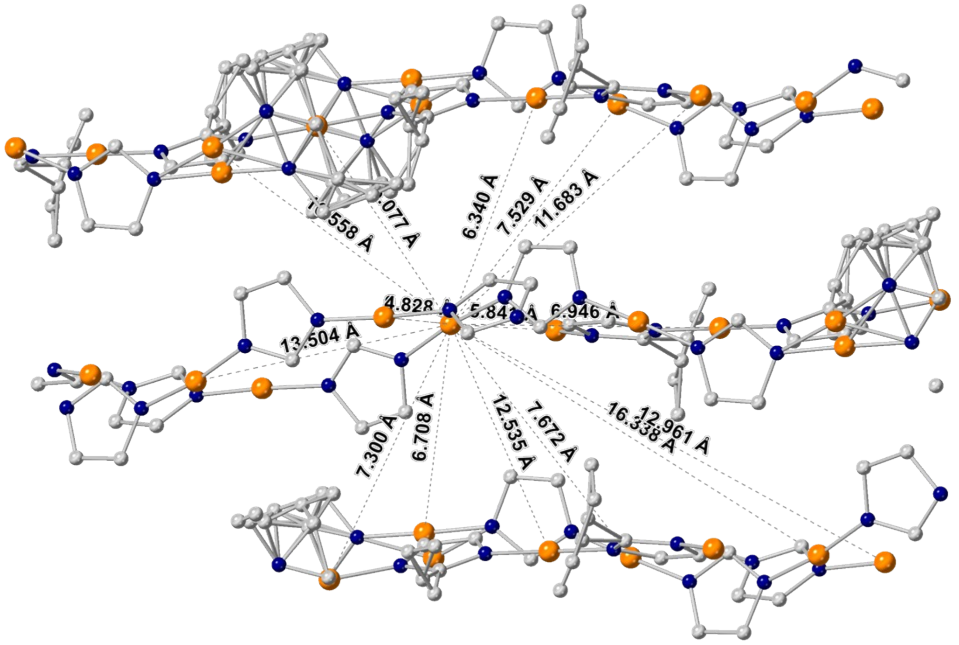

Figure S5. Selected representation of interchain Cu-Cu distances of **1** in the single crystal X-ray structure. Isopropyl groups are omitted.

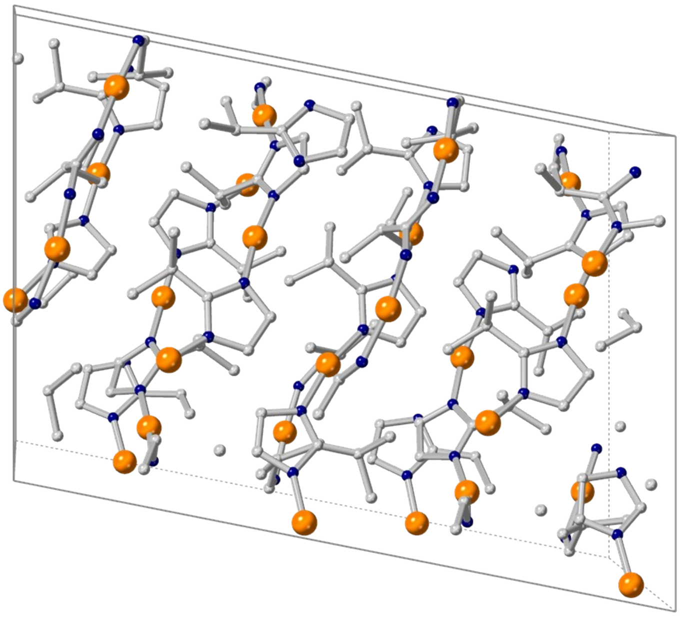

Figure S6. Structure of **1** at 25 °C by MD simulation. Cu, orange; C, grey; N, blue. Hydrogen atoms were omitted.

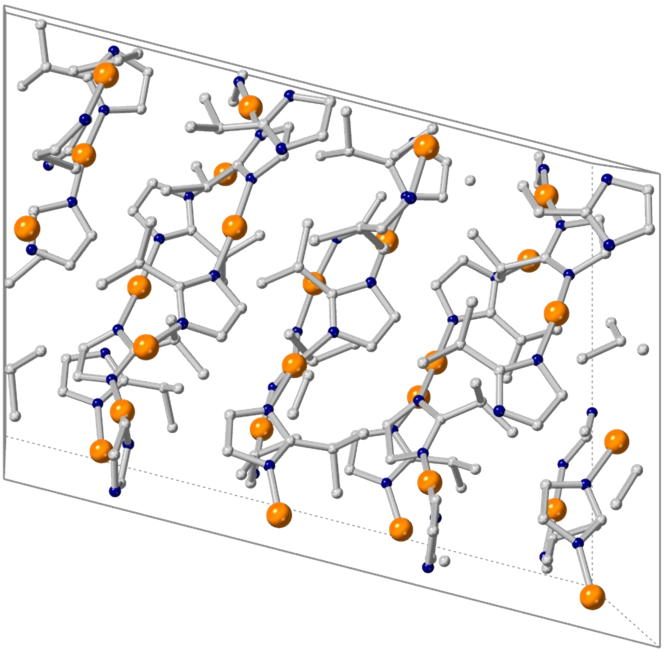

Figure S7. Structure of **1** at 175 °C (i.e. **1-melt**) by MD simulation. Cu, orange; C, gray; N, dark blue. Hydrogen atoms were omitted.

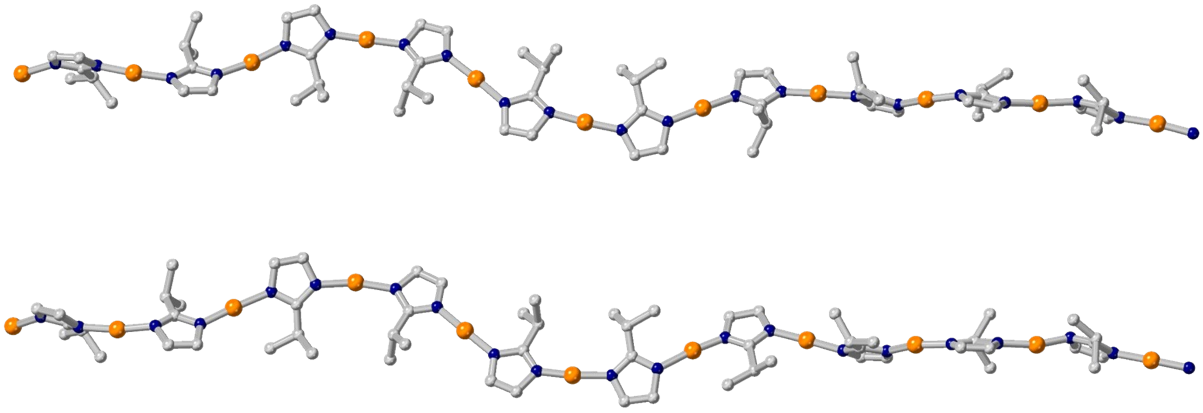

Figure S8. Comparison of the chain structures of **1** at 25 °C (top) and **1-melt** at 175 ºC (bottom). Hydrogen atoms were omitted.

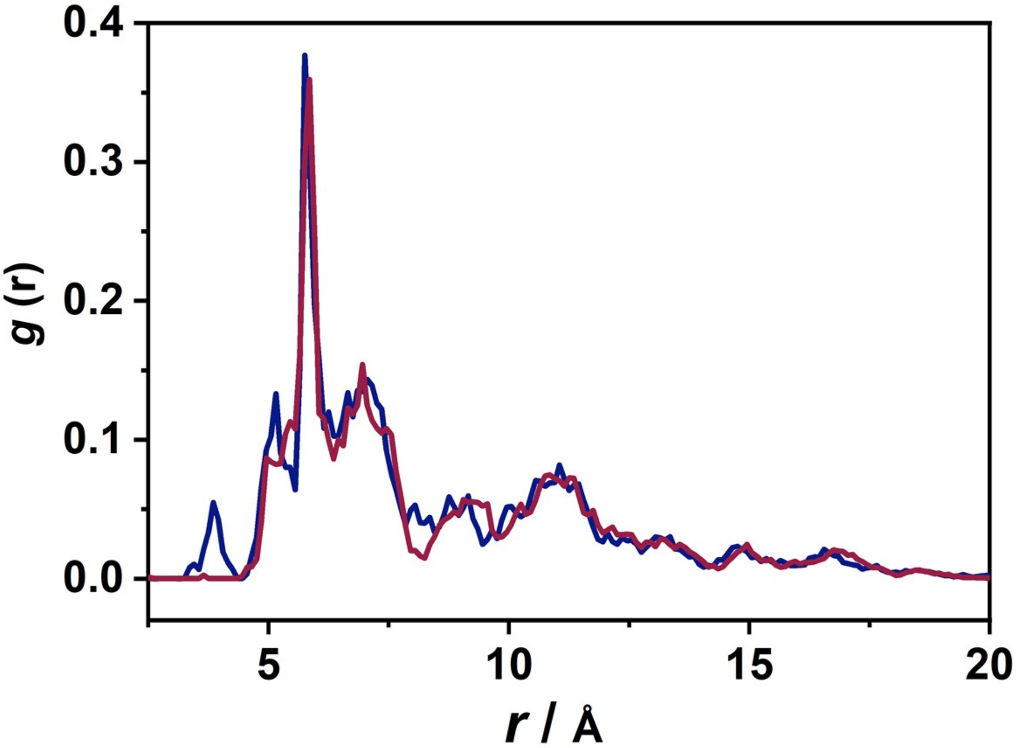

Figure S9. Calculated Cu-Cu pair correlation function of **1** at 25 °C (blue) and **1-melt** at 175 °C (red) from MD calculations.

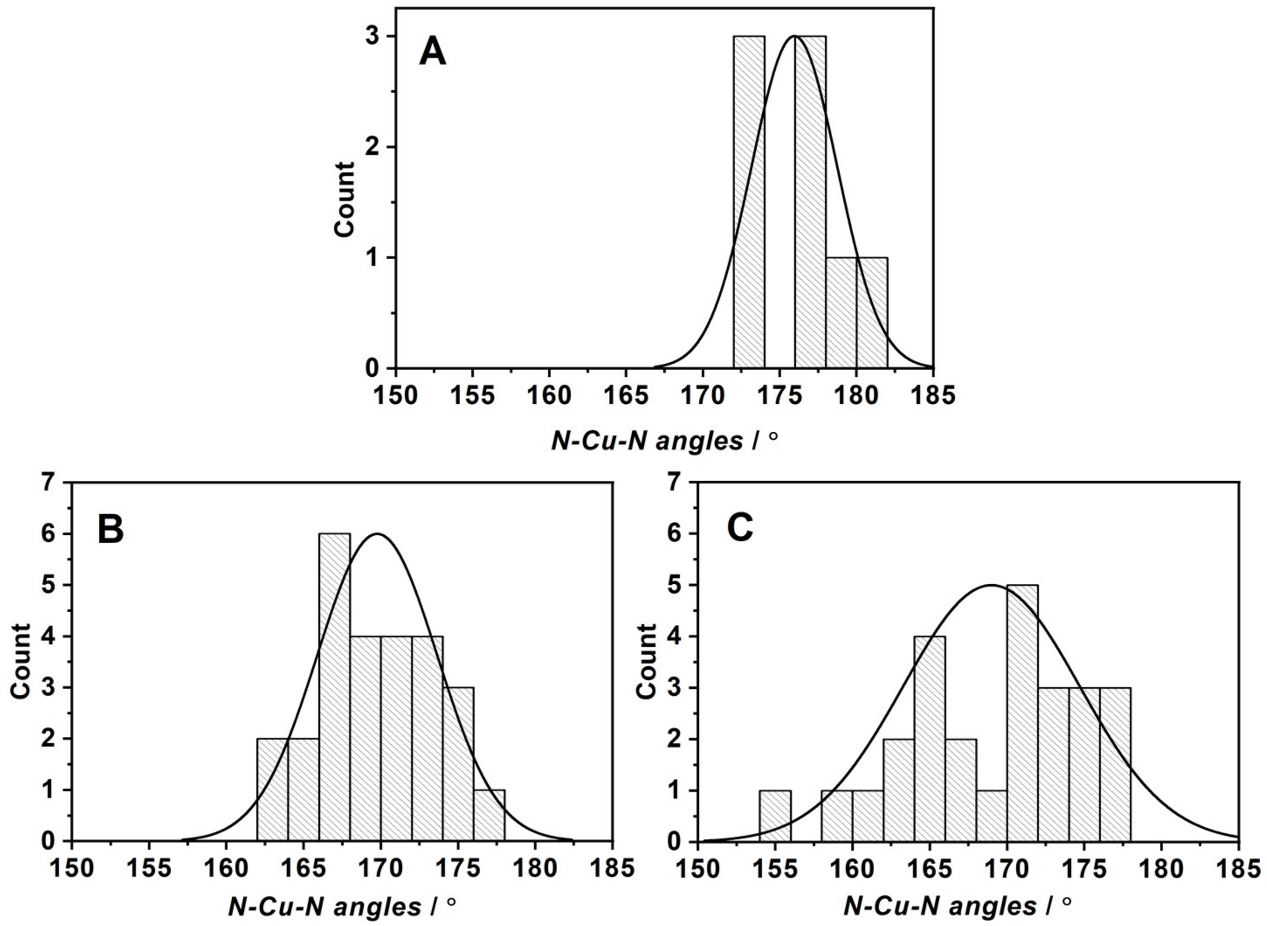

Figure S10. (A) Distribution plot of N-Cu-N bond angles of **1** from single crystal (B) **1(model)** and (C) **1-melt(model)** from MD calculations.

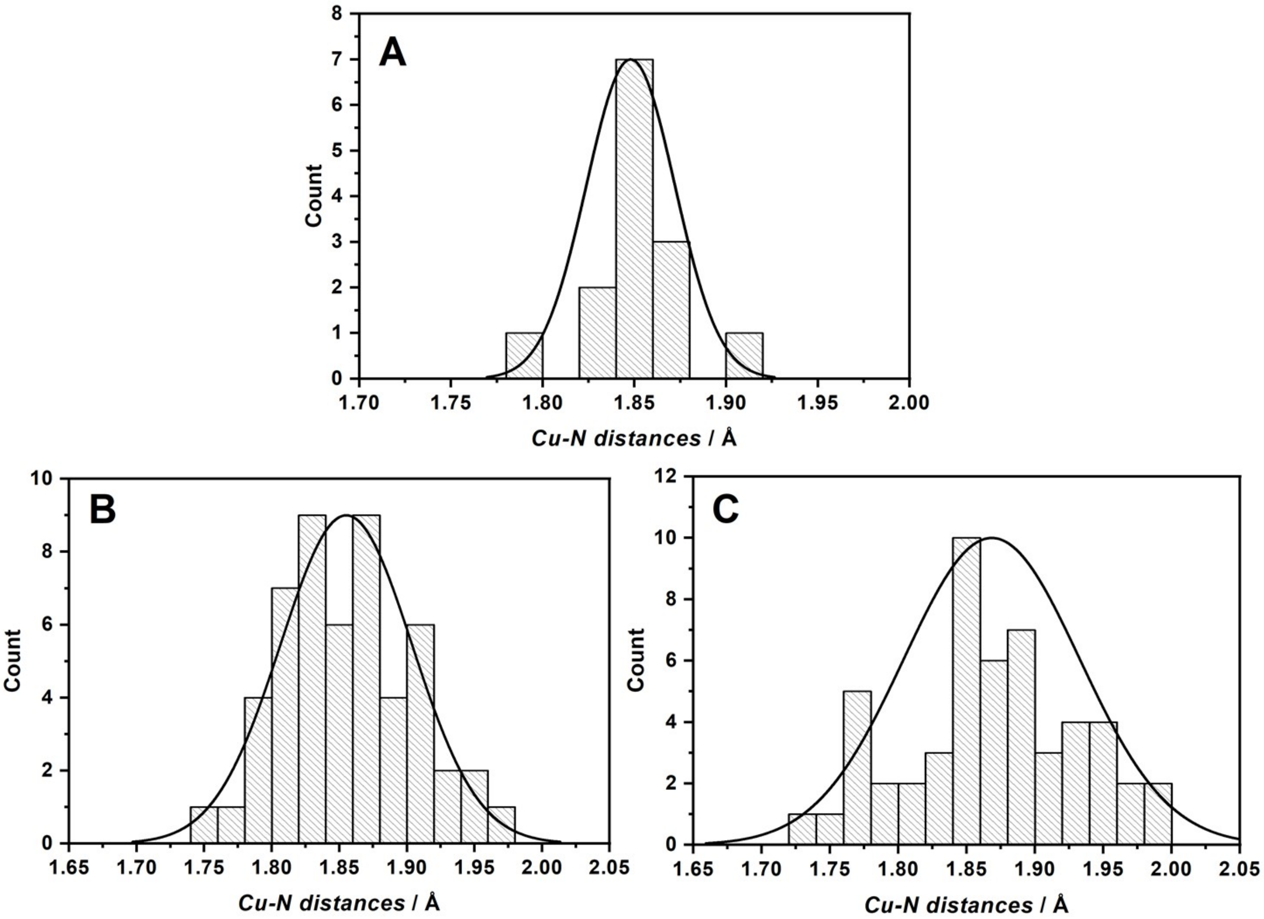

Figure S11. (A) Distribution plot of Cu-N distances of **1** from single crystal (B) **1(model)** and (C) **1-melt(model)** from MD calculations.

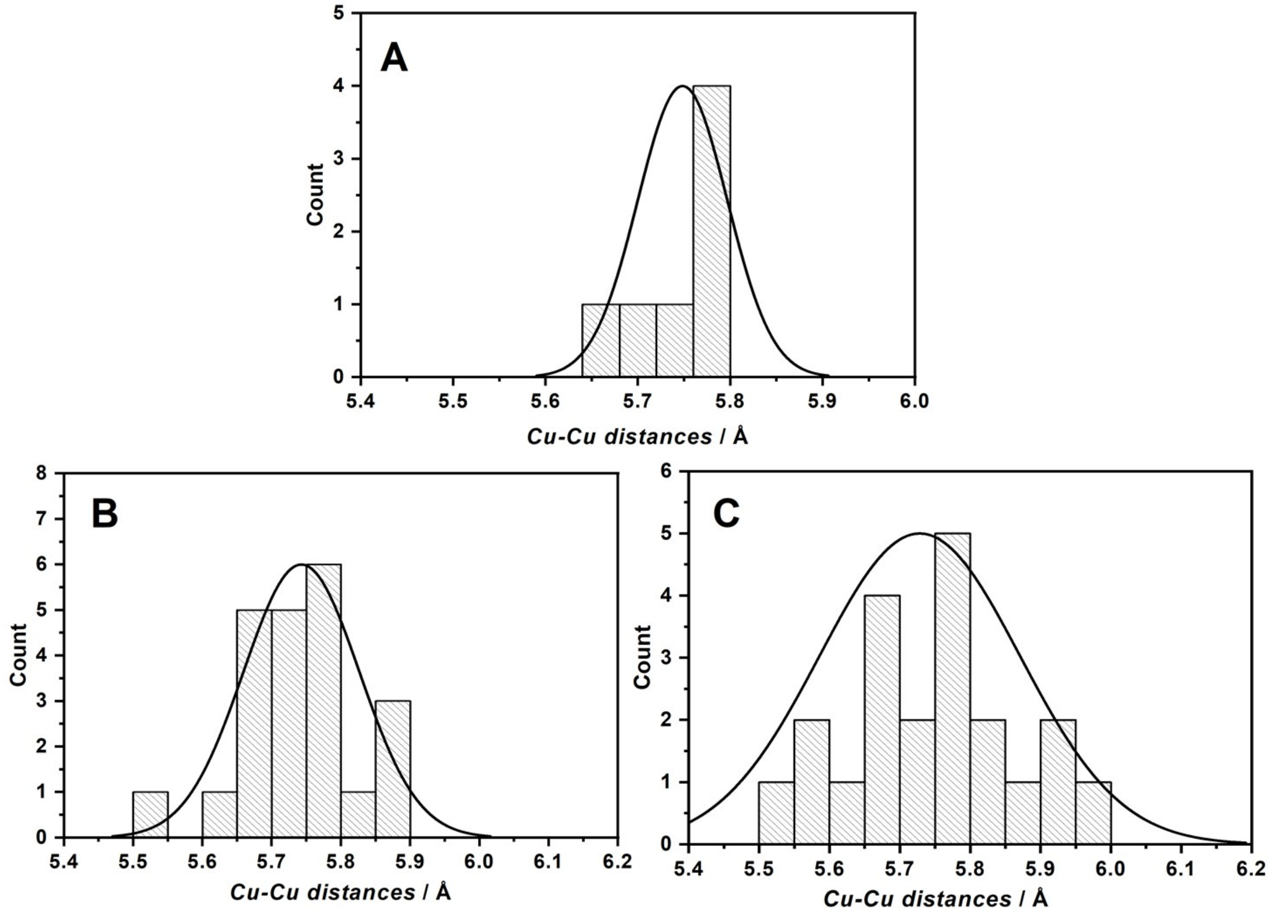

Figure S12. (A) Distribution plot of intramolecular Cu-Cu distances in the chains of **1** from single crystal (B) **1(model)** and (C) **1-melt(model)** from MD calculations.

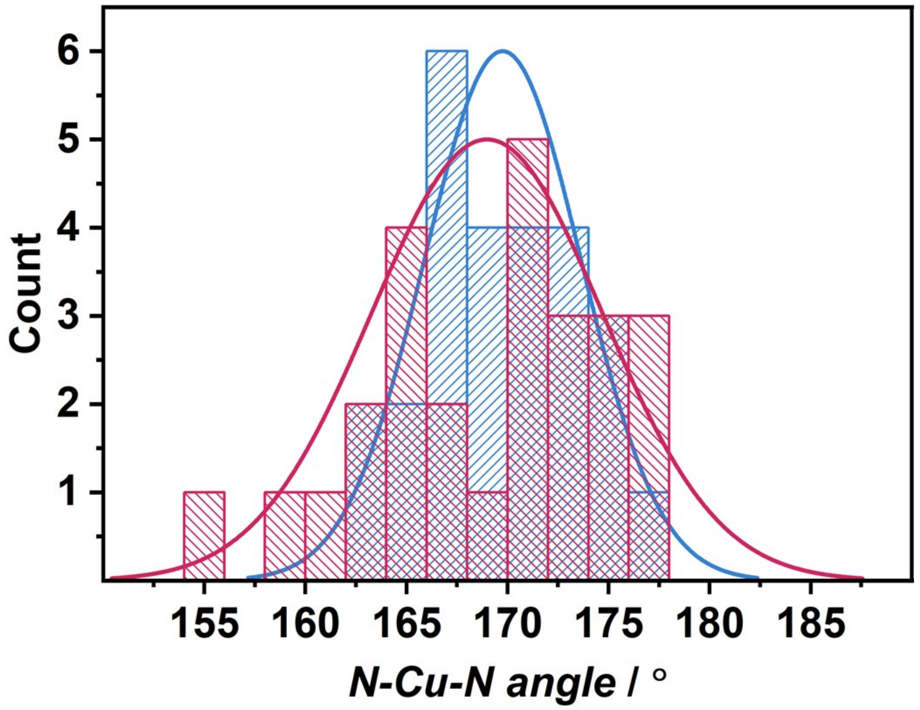

Figure S13. Distribution plot of N-Cu-N angles of **1(model)** (blue) and **1-melt(model)** (red) generated from MD simulation.

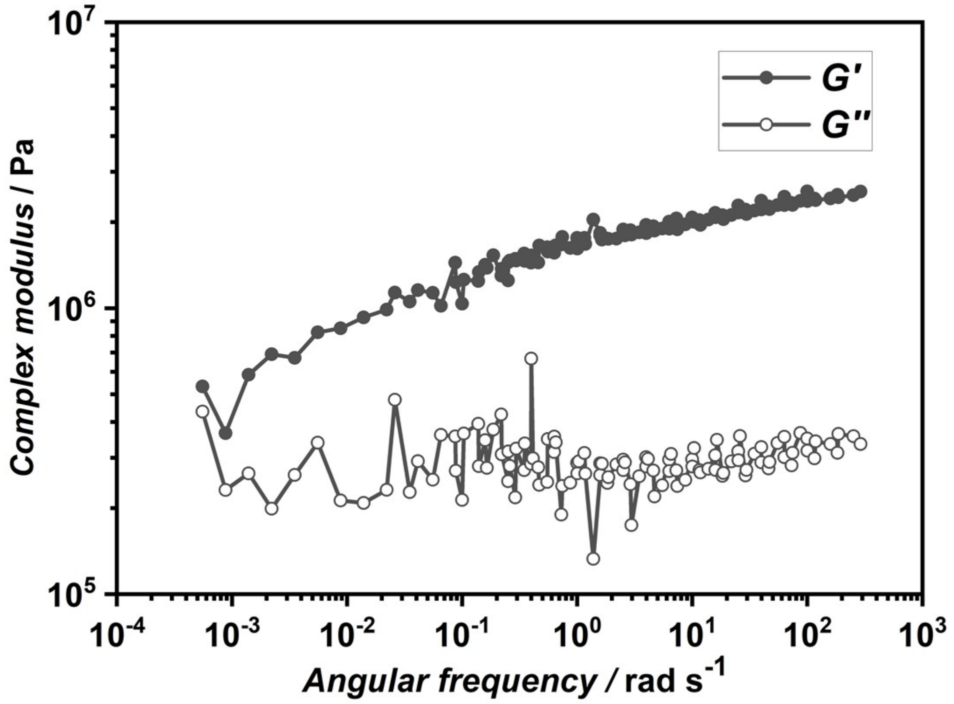

Figure S14. Time–temperature superposition master curves of **1** at 235, 215, 195, 175, 155, 135, 115 °C. The master curve obtained from Time Temperature Superposition principle by oscillatory shear rheology measurements using 175 °C as a reference temperature.

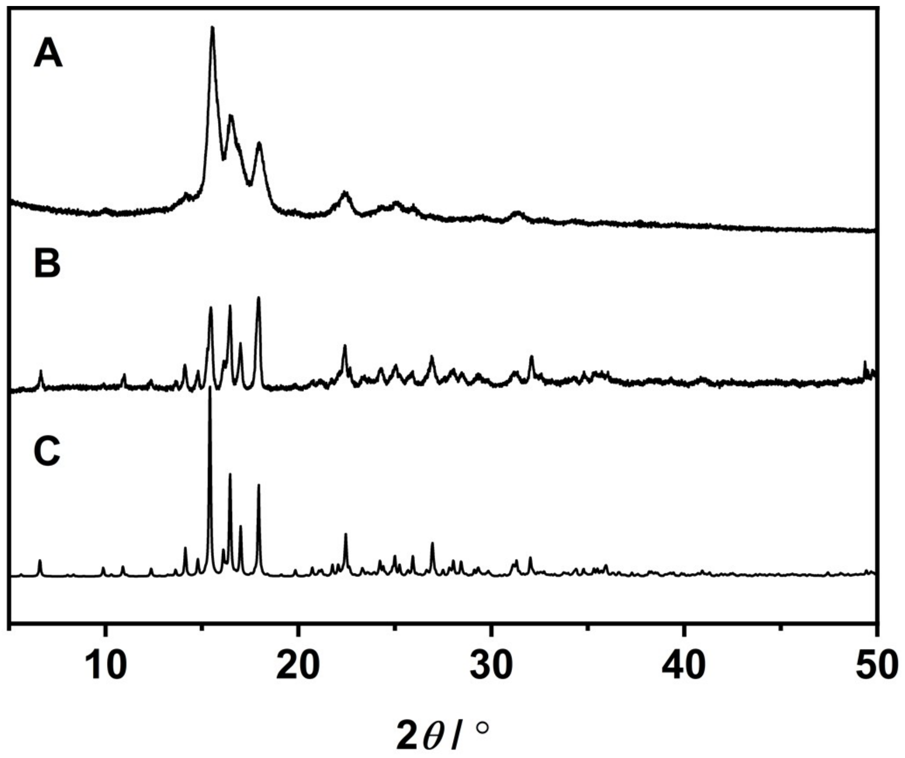

Figure S15. PXRD patterns of (A) fibrous-shaped **1** shown in Figure 3C, (B) as-synthesized **1**, (C) simulation from the single crystal X-ray structure of **1**.

Table S1. Summary of the EXAFS fitting parameters to observe the coordination number (CN) of **1** and **1-melt**.

**Sample**	***Temperature*** **/ °C**	***CN***	***R*** **(Å)**	**ΔE_0 (eV)**	***R*-Factor**	***SD*** **(+/-)**
**1**	25	2.00	1.861	6.660	0.040	-
**1-melt**	175	1.97	1.862	7.648	0.030	0.49
**1-melt**	300	1.79	1.865	7.786	0.027	0.41
**1**	90 (cooling)	1.87	1.867	8.098	0.034	0.18
**1**	25 (cooling)	1.98	1.863	7.849	0.044	0.12

S_0^2 is the amplitude reduction factor (0.863); R is the bond distance of Cu-N; ΔE_0 is edge-energy shift (the difference between the zero kinetic energy value of the sample and that of the theoretical model); R factor is used to value the goodness of the fitting; SD is the standard deviation of CN; Fourier transformation was *k*3-weighted in the *k* range of 3.0 to 8.0 $Å^{-1}$; Fitting range: 1 to 4 Å.

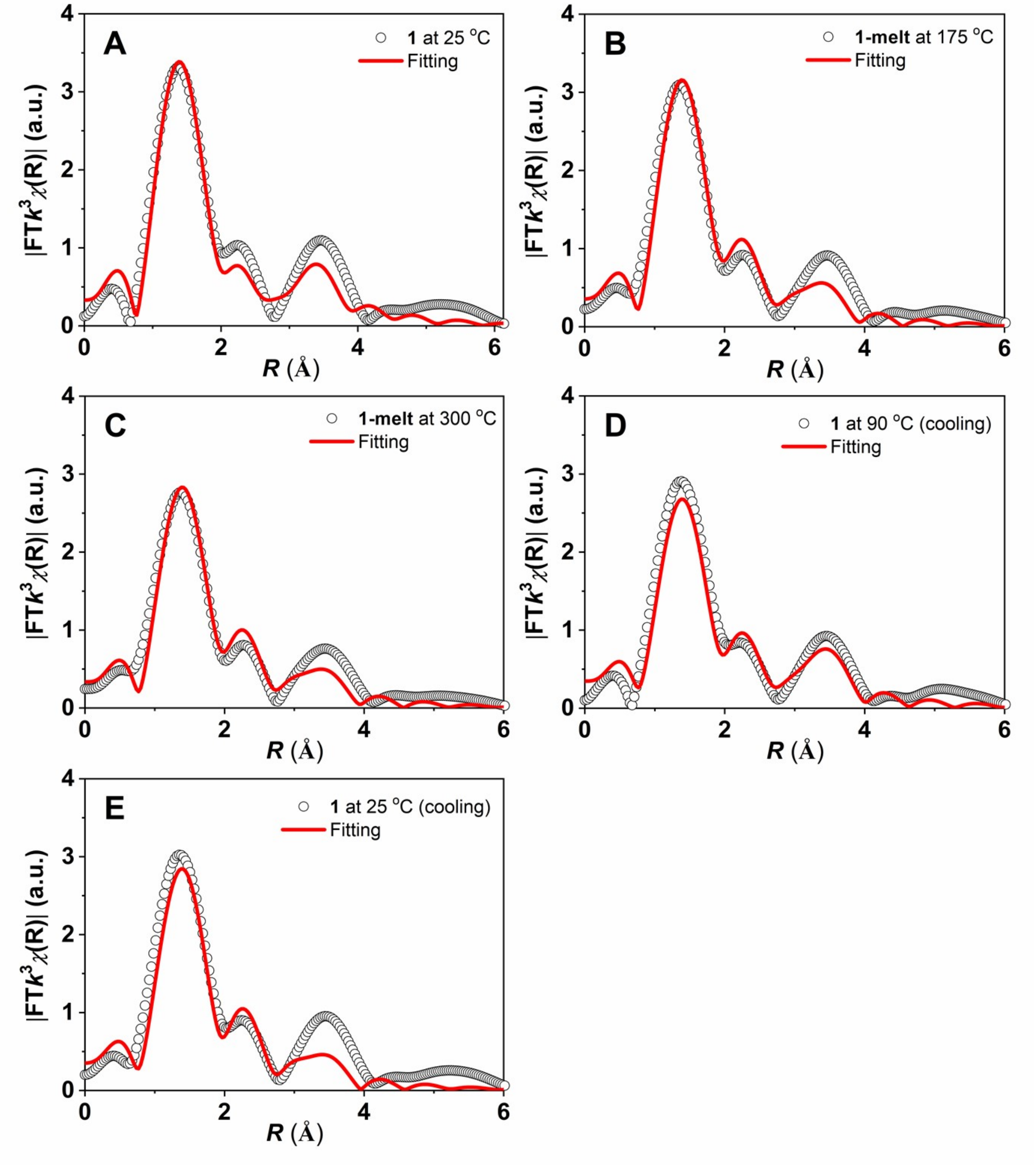

Figure S16. Radius distribution functions and the fitting results of (A) **1** at 25 °C (B) **1-melt** at 175 °C (C) **1-melt** at 300 °C in the heating process, and (D) **1** at 90 °C (E) **1** at 25 °C in the cooling process.

REFERENCES

1. Su, Y.-J.; Cui, Y.-L.; Wang, Y.; Lin, R.-B.; Zhang, W.-X.; Zhang, J.-P.; Chen, X.-M., Copper(I) 2-Isopropylimidazolate: Supramolecular Isomerism, Isomerization, and Luminescent Properties. *Cryst. Growth. Des.* **2015**, 15 (4), 1735-1739.
2. Schroter, K.; Hutcheson, S. A.; Shi, X.; Mandanici, A.; McKenna, G. B., Dynamic shear modulus of glycerol: Corrections due to instrument compliance. *J. Chem. Phys.* **2006,** *125* (21), 214507.
3. Hutcheson, S. A.; McKenna, G. B., The measurement of mechanical properties of glycerol, m-toluidine, and sucrose benzoate under consideration of corrected rheometer compliance: An in-depth study and review. *J. Chem. Phys.* **2008,** *129* (7), 074502.
4. B. Ravel and M. Newville, *J. Synchrotron Radiat.* 2005, **12**, 537-541.
5. Kresse, G.; Furthmuller, J., Efficiency of ab-initio total energy calculations for metals and semiconductors using a plane-wave basis set. *Comp. Mater. Sci.* **1996,** *6* (1), 15-50.
6. Kresse, G.; Furthmüller, J., Efficient Iterative Schemes for Ab Initio Total-energy Calculations Using A Plane-wave Basis Set. *Phys. Rev. B* **1996,** *54* (16), 11169-11186.
7. Perdew, J. P.; Ernzerhof, M.; Burke, K., Rationale for Mixing Exact Exchange with Density Functional Approximations. *J. Chem. Phys.* **1996,** *105* (22), 9982-9985.
8. Grimme, S., Semiempirical GGA-type density Functional Constructed with A Long-range Dispersion Correction. *J. Comput. Chem.* **2006,** *27* (15), 1787-1799.
9. Anisimov, V. I.; Zaanen, J.; Andersen, O. K., Band theory and Mott Insulators: Hubbard U Instead of Stoner I. *Phys. Rev. B* **1991,** *44* (3), 943-954.
10. Mueller, T.; Hautier, G.; Jain, A.; Ceder, G., Evaluation of Tavorite-Structured Cathode Materials for Lithium-Ion Batteries Using High-Throughput Computing. *Chem. Mater.* **2011,** *23* (17), 3854-3862.
11. Humphrey, W.; Dalke, A.; Schulten, K., VMD: Visual Molecular Dynamics. *J. Mol. Graphics* **1996,** *14* (1), 33-38.
